# The different expressed serum proteins in rhCygb treated rat model of liver fibrosis by the optimized two-dimensional gel electrophoresis

**DOI:** 10.1371/journal.pone.0177968

**Published:** 2017-06-29

**Authors:** Gaotai Cai, Bohong Chen, Zhen Li, Wei Wei, Ping Wang, Wenqi Dong

**Affiliations:** 1 Department of Laboratory Medicine and Biotechnology, Southern Medical University, Guangzhou, Guangdong Province, PR China; 2 School of Life Sciences, Sun Yat-sen University, Guangzhou, Guangdong Province, PR China; Istituto di Ricovero e Cura a Carattere Scientifico Centro di Riferimento Oncologico della Basilicata, ITALY

## Abstract

Liver fibrosis, a common pathological process of chronic liver diseases, is the final stage of liver dysfunction that has severely deleterious impact on human health. Cytoglobin was first discovered in 2001 by proteomic analysis in rat stellate cells and was reported to play an important role in controlling tissue fibrosis. However, the mechanism by which cytoglobin inhibits or reverses the progression of fibrosis remains unclear. The present study examines the effect of recombinant human cytoblobin (rhCygb) in a rat model of liver fibrosis. Proteomic approaches were employed to identify differentially expressed proteins in the fibrosis model. Optimized conditions for two-dimensional gel electrophoresis were developed to provide improved protein detection and separation. A total of 43 spots were obtained and, through the use of matrix-assisted laser desorption ionization time-of-flight mass spectrometry, 30 differentially expressed proteins were identified. Gene ontology term annotation and KEGG pathway analysis allowed us to explore the function of the represented proteins. Based on these results, we provide a theory of the molecular mechanism related to rhCygb reversion of fibrosis and which will assist in the identification of biomarkers in patient serum to improve early diagnosis of liver fibrosis.

## Introduction

Liver fibrosis is a response to chronic liver injury and is caused by a series of actors[1, 2]. It is well-established that liver fibrosis is a total pathological change induced by sustained liver injury, liver cell necrosis or inflammation. The underlying pathological process involves the activation of quiescent hepatic stellate cells (HSCs) into contractile myofibroblast-like cells, which secrete excessive extracellular matrix (ECM) proteins, including collagen, in the liver[3]. The inflammation resulting from liver fibrosis is difficult to repair and may ultimately lead to liver disease or cancer. Therefore, any reversal of advanced liver fibrosis prior to cirrhosis would be of great importance to human health[4].

Cytoglobin (Cygb) is a hexacoordinated heme-containing globin, identified in recent years[5], located in the fibroblast cytoplasm and nucleus of most visceral tissue. As a member of the globin family[6], which includes myoglobin, hemoglobin, and neuroglobin, Cygb has the same function of binding and transporting oxygen. In particular, Cygb is expressed ubiquitously in the cytoplasm of pericytes in many organs, including the brain, thymus, heart, lung, liver, kidney, small intestine and spleen[7]. One interesting aspect of Cygb expression is its presence in visceral cells that have the ability to store vitamin A. It has been reported that Cygb protects cells from reactive oxygen with its functions of O2 storage, diffusion and sensing for cellular respiration and metabolism, NO scavenging and involvement in hypoxia and oxidative stress[8–10]. These findings prompted the hypothesis that Cygb could be effective in the prevention or treatment of liver fibrosis[11]. The molecular mechanism for this hypothesis is still not clear, but its nitrogen oxide dioxygenase activity and lipid peroxidase activity have been demonstrated[10].

Recently, proteomic approaches have been utilized to identify protein markers for specific diseases or disease stages[12]. Liver biopsy remains the gold standard by which to assess hepatic fibrosis, but it is invasive, expensive and often poorly tolerated by patients[13]. For these reasons, it is desirable to predict hepatic fibrosis without invasive liver biopsy. An easily obtained sample of serum or plasma is the promising choice for biomarker studies[14]. Proteomic techniques allow unbiased assessment of proteins and may be useful to identify proteins related to hepatic fibrosis. Two-dimensional gel electrophoresis (2-DE) is a common approach for proteomic analysis which separates and identifies hundreds of proteins[15–17]. However the proteomic analysis of serum samples remains a challenge due to the presence of a few highly abundant proteins, such as albumin and immunoglobulins. Removal of uninformative, high expression proteins is desired to enhance the sensitivity of analysis to the disease related protein of interest[18–20]. In this study, rat serum was pretreated with a 2DE Clean-Up kit to remove interfering components, such as high concentrations of salt ions, lipids, and polysaccharides. The samples were further refined using a ProteoMiner kit, employing a large bead-based library of combinatorial peptide ligands, to dilute high-abundance proteins and concentrate low-abundance proteins, or an Albumin/IgG removal kit. A comparison of the effectiveness of these protein isolation and enhancement technologies allowed us to optimize pre-treatment conditions for 2-DE. Finally, the serum samples were analyzed by 2-DE to identify a protein panel of possible biomarkers in the liver fibrosis model.

## Materials and methods

### Reagents

Aspartate aminotransferase (AST) and alanine aminotransferase (ALT) were purchased from Nanjing Jiancheng Institute of Biotechnology (Nanjing, China). Hyaluronic acid (HA), laminin (LN), collagen III (Col III) and collagen IV (Col IV) ELISA kits were obtained from Abbott Laboratories, USA. A Bradford kit was bought from Sangon Biotech, Shanghai, China. Sirius red and hematoxylin-eosin (HE), protease inhibitor cocktail, 3-[(3-Cholamidopropyl) dimethylammonio]-propanesulfonate (CHAPS), glycine, ammonium persulfate (APS), TEMED, and protease inhibitor cocktail were obtained from Sigma Chemical (St. Louis, MO, USA) Dithiothreitol (DTT), glycine, Immobilized pH gradient (IPG) strips, IPG buffer, dry strip cover fluid and other reagents that were used in two-dimensional gel electrophoresis were acquired from Bio-Rad (Hercules, CA, USA).

### Animals and treatments

Sexually mature Sprague-Dawley (SD) rats (n = 90), weighing 180–220 g, were obtained from the Experimental Animal Center of Southern Medical University, Guangzhou, China. Approval of Southern Medical University Animal Care and Use Committee (IACUC) Number is 2014–025 and Permit Number is SCXK20110015. Care of the animals used in this investigation was conducted according to the guidelines approved by the Chinese Association of Laboratory Animal Care. Rats were anesthetized with intraperitoneal injections of 2% sodium pentobarbital (50mg/kg body weight) and sacrificed with the theoretical injection of 1.2 to 1.5 times dose according to Southern Medical University IACUC-approved procedures. They were housed in a room under a constant temperature (20±2°C) and humidity (70%) with a 12 h light/dark cycle. The rats had free access to standard diet and water. The animals were randomly assigned to two groups, the control group (n = 30) and the carbon tetrachloride (CCl4) induced hepatic injury group (n = 60). A rat model of liver fibrosis was created using two treatment groups: a mild model group and medium model group. The mild model group was treated twice per week for 10 weeks with intraperitoneal injections of 25% CCl4 in paraffin oil at 2 ml/kg. The rats in the moderate liver fibrosis model group were treated as in the mild model group, but received additional treatment for 3 weeks of intraperitoneal injections of 50% CCl4 twice per week, adjusting the dose for the weight of each rat. Serum levels of ALT, AST, HA, Col III, and Col IV were measured in the two groups. Staining with Sirius red and HE confirmed that CCl4 treatment successfully induced hepatic fibrosis.

After the fibrosis model was established, liver fibrosis model rats (n = 30) were treated daily with rhCygb (2 mg/kg body weight/day) and twice per week with 25% CCl4. The production and purification of rhCygb were prepared by our laboratory[11]. Rats were anesthetized and sacrificed within 24 h after the last treatment. Histological examination of the liver and serum parameters were evaluated as before. Blood samples were collected from the abdominal aorta. The serum was separated via centrifugation at 5000rpm for 10 minutes at 4°C and stored at −80°C for further study.

### Optimizing methods for 2-DE

Serum samples were obtained from the control group, CCl4 model group and rhCygb treated group. We explored different kits for pre-treatment and different ranges of IPG strips for 2-DE. The interfering components (high concentrations of salt ions, lipids, and polysaccharides) in the serum supernatant were removed using a 2-D Clean-Up Kit. Further refining of the samples was done with the ProteoMiner kit and compared against samples treated with an Albumin/IgG removal kit in order to provide a valuable tool for enhancing the detection of low-abundance proteins. Briefly, a columnfrom the ProteoMiner Protein Enrichment Kit was loaded with 1ml of serum sample containing a protease inhibitor cocktail and incubated for two hours at room temperature under constant rotation. After incubation, the column was washed 3 times with 500μL of PBS to remove the unbound high-abundance proteins. The desired proteins were eluted from the column with a 5% acetic acid buffer (3 times) and collected. Also the Albumin/IgG removal kit was carried out according to the manufacturer's instructions Protein was measured with a Bradford kit. A total of 120μg of protein in 300μL 2D sample rehydration buffer was separately loaded onto a 17cm linear IPG strip (pH 5–8) and 17cm nonlinear IPG strip (pH 3–10) for first-dimension isoelectric focusing (IEF). The strips were placed into a Protean IEF cell and rehydrated at 50 volts for 16 hours. IEF was carried out at 250 volts for 1 hour, 500 volts with rapid climbing for 1 hour, 1000 volts with rapid climbing for 1 hour and a final linear rapid voltage gradient to 10,000 volts until reaching 65000 volt-hour (current limit was set at 50μA at 20°C). Focused strips were held under 500 volts until ready for equilibration. After IEF, the IPG strips were blotted against damp filter paper to remove excess mineral oil and then were equilibrated for 15 minutes in a buffer containing 50mM Tris-HCl, pH8.8, 20% glycerol, 6M urea, 1% sodium dodecyl sulfate (SDS), and 2% DTT. The strips were further treated in a similar buffer (containing 2.5% iodoacetamide) for 15 minutes and washed with SDS electophoresis buffer before being directly applied into 12.5% homogeneous SDS-PAGE gels. The gels were run in parallel at 10mA for 60 minutes, and then run at 28mA until the bromophenol blue dye reached the bottom. Each experiment was performed in triplicate.

### Image analysis and protein identification

Two dimensional gels were stained with the silver method to make it compatible with mass spectrometry. The stained gels were scanned using UMax Magic scan (Amersham Biosciences, Sweden), and analyzed with PDQuest 8.0.1 (Bio-Rad). In-gel digestion and matrix-assisted laser desorption ionization time-of-flight mass spectrometry (MALDI-TOF-MS) for identification of different proteinsanalysis were performed at the Beijing Genomics Institute in Shenzhen, China. Combined peptide mass fingerprinting (PMF) and tandem mass spectrometry (MS/MS) queries were searched against the NCBI database, using the MASCOT search engine from Matrix Science (http://www.matrixscience.com/) to identify the proteins. Proteins matching more than five peptides and with a MASCOT score higher than 60 were considered significant (p < 0.05).

### Protein interaction analyses and bioinformatics analysis

The web portal for the STRING database (http://www.string-db.org/) was used for protein—protein interaction (PPI) network analysis. The networks that involved proteins derived from 2-D DIGE experimentations 30 differentially expressed and identified proteins, and architecture represents connections between the individual proteins. To examine the functions and pathways of the differentially expressed proteins, we searched the UNIPROT database (http://www.uniprot.org/) to identify the functions and relevant pathways of these proteins. To functionally annotate the correlated genes, gene ontology (GO) and pathway analysis was conducted using the Database for Annotation, Visualization and Integrated Discovery (DAVID) functional annotation tool (http://david.ncifcrf.gov/tools.jsp). Pathway enrichment analysis of the differentially expressed proteins was obtained from the KEGG database.

### Statistical analysis

The Statistical Program for Social Sciences (SPSS) version 19 was used for data analysis calculations. All data are expressed as the mean ± standard error of the mean. The Levene test for homogeneity test of variance and One-way ANOVA was used for comparison among groups. Differences between two groups were compared using an unpaired Student t-test. Values of p< 0.05 were considered statistically significant.

## Results

### CCl_4_ induced hepatic fibrosis in rats

**Fig 1:**
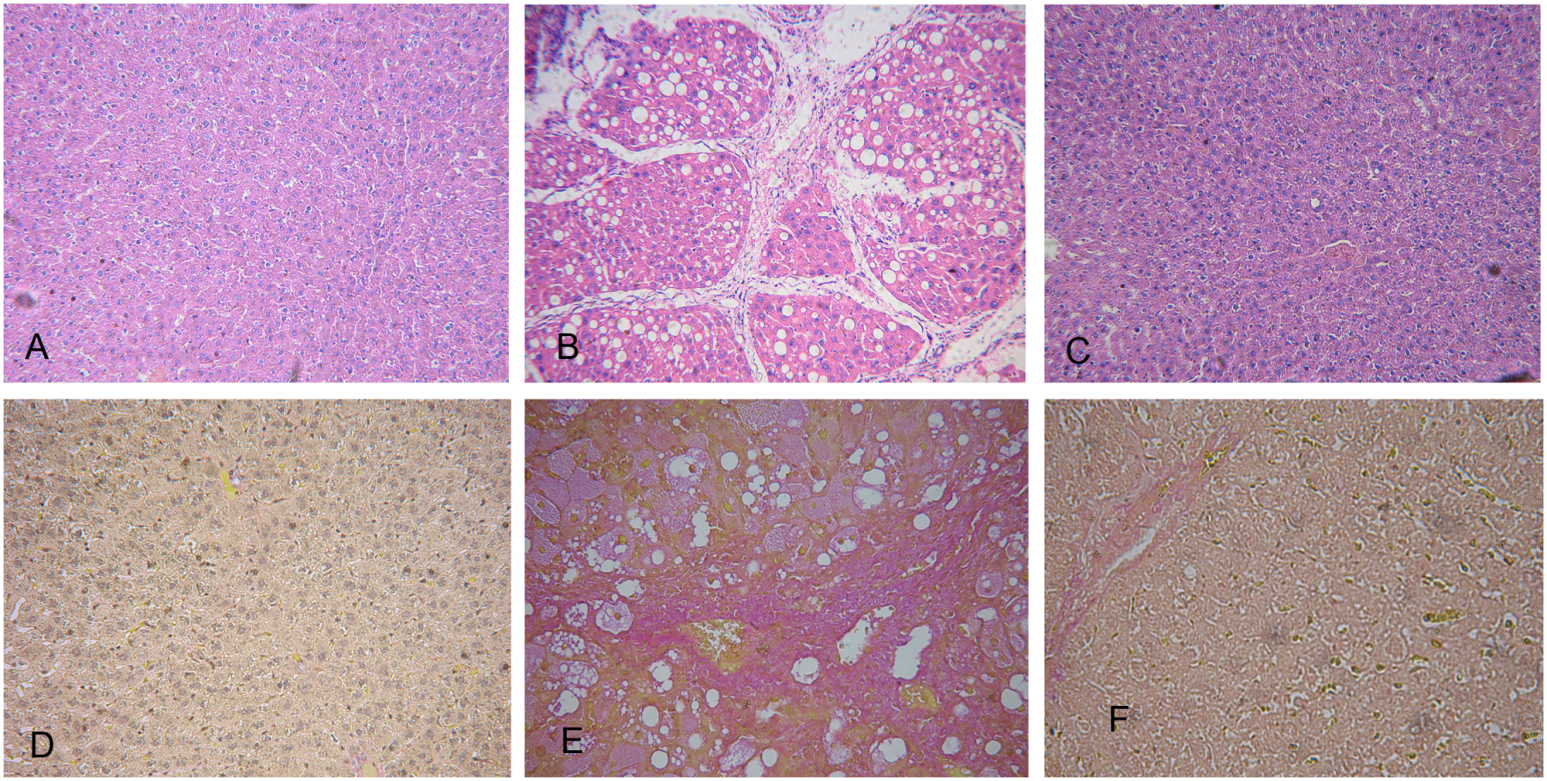
HE and Sirius red staining of liver biopsy samples among the different groups. A, B and C: the HE staining of liver biopsy of the control group, fibrosis model group and rhCygb treatment group, respectively. D, E and F: the Sirius red staining of liver biopsy samples of the control group, fibrosis model group and rhCygb treatment group, respectively.

After HE and Sirius red staining, livers from the control group showed normal lobular architecture with central venous and hepatic cord radiation (Fig 1A and 1D). The CCl4 treated group showed extensive disruption of the liver architecture, including hepatocellular necrosis and inflammatory cell infiltration (Fig 1B and 1E). Analysis of serum biochemistry (Table 1)showed that ALT and AST levels were increased (P<0.01) in the fibrosis model group. Serum LN, HA, Col III and Col IV levels in the CCl4 treated groups were higher than in the control group. These results clearly illustrate that the model of CCl4-induced fibrosis was successfully established. Meanwhile, the rhCygb treatment group displayed markedly decreased serum levels of these markers, compared with the fibrosis model group. The livers of rats treated with rhCygb displayed lower content of collagen fibers and exhibited a clear improvement when compared with the CCl4 treated group (Fig 1C and 1F).

**Table 1:** Anti-hepatic fibrosis effect of rhCygb in CCl_4_-induced liver fibrosis model rats($\bar{x}$ ± s, n = 30).

Groups	ALT(U/L)	AST(U/L)	LN(ng/ml)	HA(ng/ml)	Col III(ng/ml)	Col IV(ng/ml)
Control	35.52±4.3	37.58±6.35	26.45±6.35	97.33±15.26	14.3±3.5	26.35±5.23
Model	521.46±98.77^a^	622.33±98.77^a^	103.75±12.44^a^	378.95±48.23^a^	78.23±14.6^a^	58.78±7.89^a^
rhCygb	47.25±7.6^c^	55.26±7.6^c^	28.52±7.6^c^	108.56±25.68^c^	17.2±3.3^c^	28.24±5.66^c^

^a^ Compared with control group P<0.01;

^c^ Compared with model group P<0.01

### 2-DE pattern

**Fig 2:**
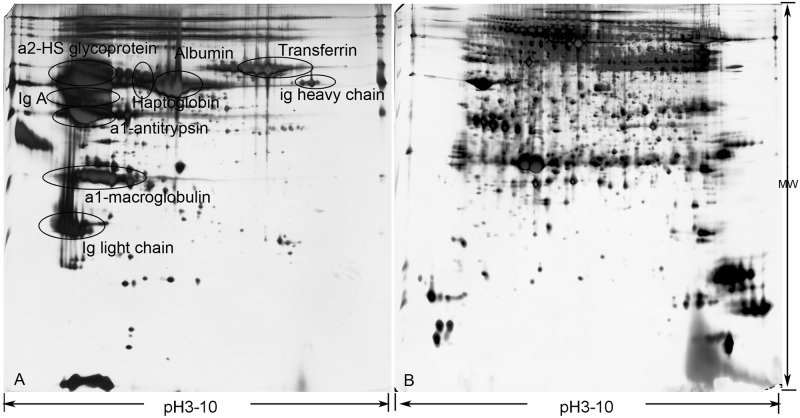
Effect after treatment with different kits on 2-DE of serum proteome. A: Using the Albumin/IgG removal kit, the proteins did not separate well. The main high-abundance proteins were α2-HS glycoprotein, albumin, transferrin, IgA, haptoglobin, Ig heavy chain, α1-antitrypsin, α1-macroglobulin and Ig light chain. B: Use of the ProteinMiner Protein Enrichment Kit resulted in proteins that were separated by a large degree.

**Fig 3:**
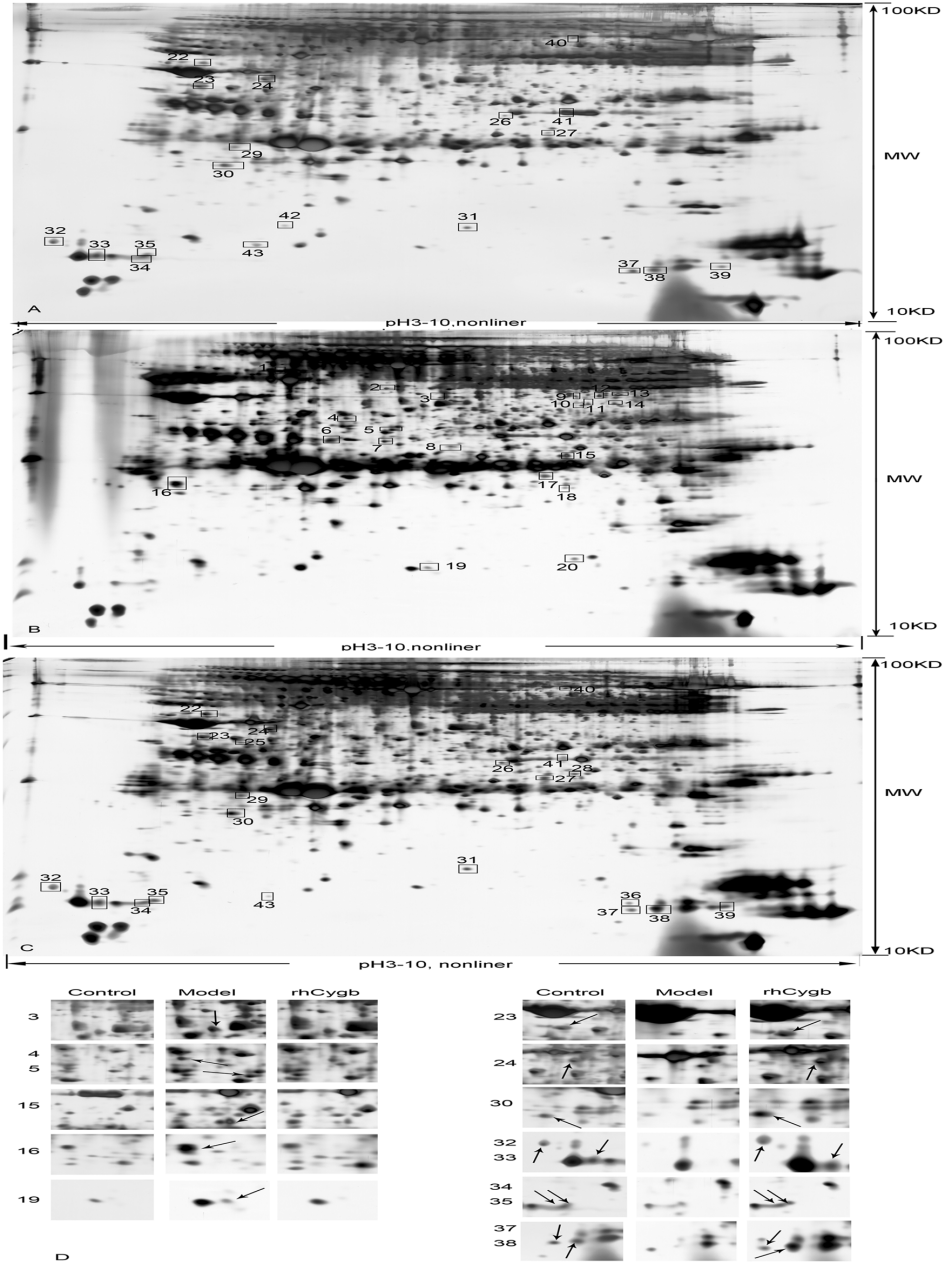
2-DE analysis of serum samples and the corresponding differentially expressed protein spots. A: Control group, B: Firbosis model group, C: rhCygb treatment group, D: Fifteen representative differentially expressed protein spots were magnified from three pairs of serum samples. The spots on the left side of the figure were up-regulated in fibrosis model group, while spots on the right were down-regulated in the fibrosis model group. Using a pH3-10, 17cm nonliner IPG Strip and 12.5%SDS-PAGE, we identified 43 different proteins on the gels.

To optimize conditions for the analysis of rat serum via 2-DE, a 2-D Clean-Up Kit was used, according to manufacturer’s recommendations, to remove interfering components and to determine the proper loading quantity of samples (120μg of protein) and the optimal 17cm nonlinear IPG strip (pH 3–10) for IEF. The ProteoMiner kit treatment showed a greater benefit for enriching low-abundance species, compared with the Albumin/IgG removal kit, as shown in Fig 2. Using 2-DE, proteins from the control, fibrosis model and rhCygb treatment groups are displayed in Fig 3. Each experiment was performed in triplicate and analyzed with PDQuest 8.0.1 software. We obtained 812 ± 28 protein spots with an 88.2 ± 7% matching rate.

### Proteins identified by MALDI-TOF-MS

Represented protein spots were excised from the 2-DE gel and analyzed for protein identification by MALDI-TOF-MS. The PMF was obtained and analyzed using the MASCOT search engine against the NCBI database. The database query resulted in 30 significant proteins and their functions, as shown in Table 2.

**Table 2:** Identification of 30 proteins differentially expressed among the control, fibrosis model, and rhCygb groups.

ID	Protein name	Swiss-prot Accession	MW	PI	Protein Score	rhCygb	Protein function
1	60 kDa heat shock protein, mitochondrial	P63039	610088.45	5.96	269	↓	Positive regulation of inflammatory response; Responseto hypoxia
2	Argininosuccinate lyase	P20673	51643.24	6.20	112	↓	Cellular response to hypoxia; Cellular response to tumor necrosis factor; Diaphragm development; Arginine biosynthetic proces process
5	FGFR1 oncogene partner 2 homolog	Q6TA25	25048.81	5.27	62.8	↓	Response to wounding; Wound healing; Accelerates the collagen gel contraction in vitro
6	Clusterin	P05371	51969.54	5.47	65.8	↓	Response to oxidative stress; Response to wounding; Positive regulation of NF-kappaB transcription factor activity
7	RGD1311188 protein	B1WC62	40258.69	9.79	62.9	↓	Metal ion binding; Nucleic acid binding
9	LRRGT00008	Q6TXJ1	183308.21	10.44	65.6	↓	RNA binding
12	Bileacid-CoA: amino acid N-acyltransferase	Q63276	46777.17	7.46	101	↓	Organ regeneration; Liver development; Bile acid metabolism
13	Pink1	D3Z9M9	27675.61	11.4	71.2	↓	Cellular response to toxic substance; Positive regulation of I-kappa B kinase/NF-kappaB signaling; Response to stress
15	Proteasome subunit alpha type-6	P60901	27837.98	6.74	72.5	↓	Positive regulation of NF-kappaB transcription factor activity; Ubiquitin-dependent protein catabolic process
16	Proteasome subunit beta type-6	P28073	25515.54	4.61	147	↓	Proteolysis involved in cellular protein catabolic process
17	Protein Smarca5	F1LNL2	15813.06	9.65	63.1	↓	ATP-binding; DNA binding binding
20	Glyceraldehyde-3-phosphate dehydrogenase	P04797	122992.34	8.4	96.4	↓	Microtubule cytoskeleton organization
23	Apolipoprotein E	P02650	103861.79	4.93	64.2	↑	Negative regulation of inflammatory response; A ligand for the LDL (apo B/E) receptor
25	Itih4	Q5EBC0	35788.35	6.11	68.3	↑	Acute-phase response; Hyaluronan metabolic process
26	NmrA-like family domain-containing protein 1	P86172	34638.68	6.8	159	↑	Redox sensor protein; Reduces the production of nitric oxide
27	Ig kappa chain C region, B allele	P01835	26017.67	5.26	78	↑	Fc-gamma receptor signaling pathway involved in phagocytosis; Innate immune response
29	Ig lambda-2 chain C region	P20767	11481.67	5.8	70.5	↑	Immunoglobulin domain
30	Peroxiredoxin-2	P35704	21941.13	5.28	160	↑	Removal of superoxide radicals; Response to oxidative stress; Peroxidase activity
31	Galectin-9	P97840	36692.36	8.66	137	↑	Ion transport
32	Galectin-5	P47967	16414.11	6.66	91.7	↑	May function in erythrocyte differentiation
33	Galectin-5	P47967	16414.11	6.66	81.6	↑	May function in erythrocyte differentiation
34	Brefeldin A-inhibited guanine nucleotide-exchange protein 2	Q7TSU1	204296.96	6.48	68.1	↑	Golgi to plasma membrane transport; Intracellular signal transduction
36	Hbb-b1 hemoglobin, beta adult major chain	Q62669	16069.28	8.51	137	↑	Oxygen transport from the lung to the various peripheral tissues
37	Beta-2-microglo-bulin	P07151	13825.17	8.23	65.7	↑	Immune response; Response to drug
38	Hemoglobin subunit alpha-1/2	P01946	15489.83	8.14	373	↑	Oxygen transport from the lung to the various peripheral tissues
39	Ifi44l	M0R4J5	90125.05	8.97	69.5	↑	Immune response
40	Hemoglobin subunit alpha-1/2	P01946	15489.83	8.14	241	↑	Oxygen transport from the lung to the various peripheral tissues
41	C4-2 complement component 4, gene 2	Q6MG90	193602.9	7.04	255	↑	Inflammatory response; Complement activation
42	Complement C4	P08649	193638.91	7.34	202	↑	Inflammatory response; Complement activation; Innate immune response
43	Complement C4	P08649	193638.91	7.34	95.9	↑	Inflammatory response; Complement activation; Innate immune response

### Protein—Protein interaction network and GO enrichment pathway analysis

**Fig 4:**
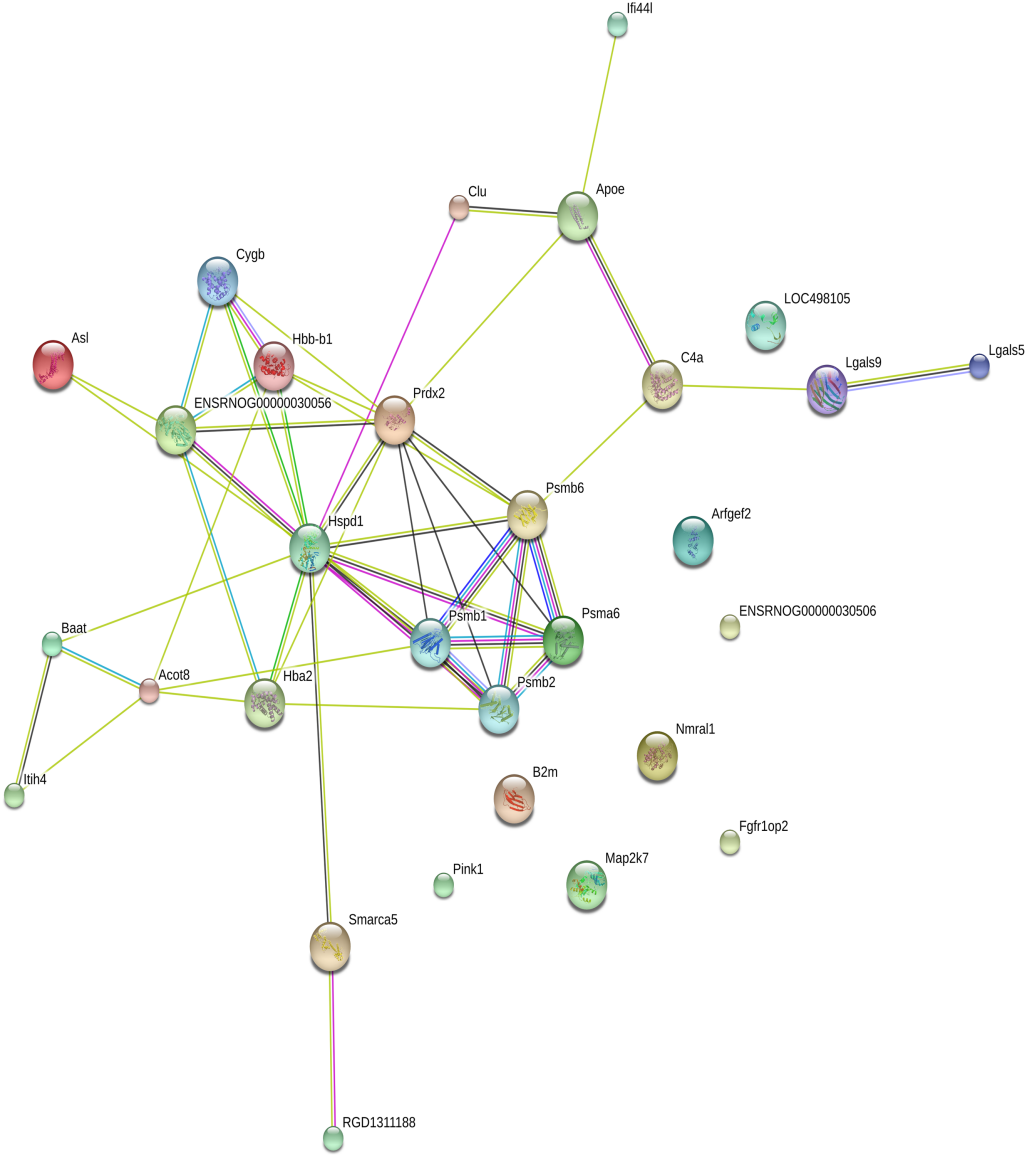
Analysis of Protein—Protein Interaction Network.

**Fig 5:**
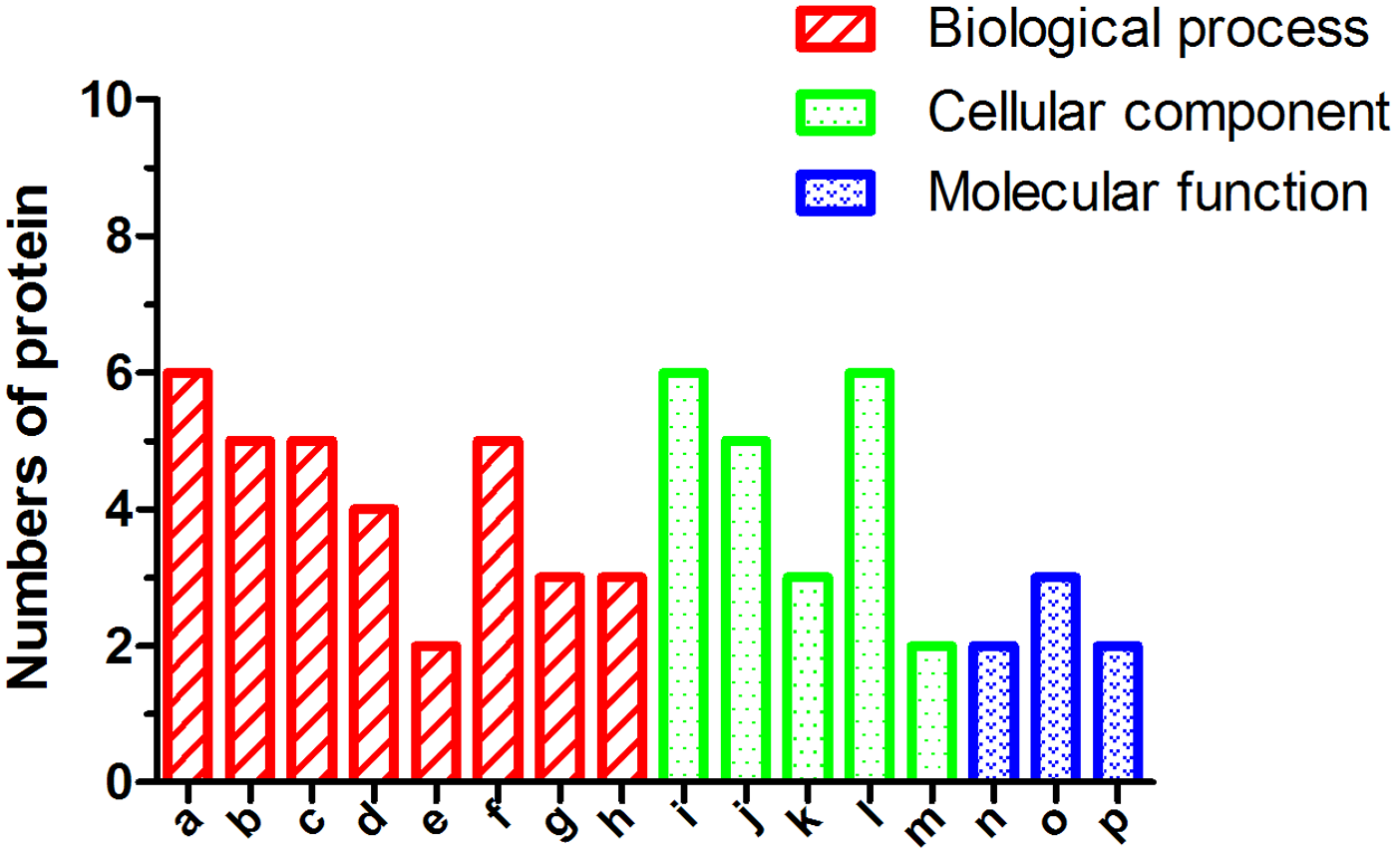
Distribution of all serum altered proteins into different functional and categories. a: response to oxidative stress; b: regulation of response to stimulus; c: regulation of immune system process; d: response to inflammatory; e: response to wounding; f: anti-apoptotic process; g: cellular macromolecule catabolic process; h: positive regulation of NF-kB transcription factor activity; i: extracellular region; j: cytosol; k: endosome; l: mitochondrion; m: plasome lipoprotein particle; n: ATP binding; o: ubiquitin protein ligase binding; p: antioxidant activity.

Information on Protein—Protein Interaction was obtained from STRING database (Fig 4). The networks that involved proteins derived from 2-D DIGE experimentations 30 differentially expressed and identified proteins. To examine the functions and pathways of the 30 up-regulated and down-regulated proteins, the enriched GO terms were categorized for liver fibrosis networks to identify their functional roles. According to the GO categories, the identified proteins could be divided into several relevant metabolic processes (Fig 5), which included responses to oxidative stress and stimulus, NF-κB signaling, catabolic processes and antioxidant activity. The differentially expressed proteins, and their functional categories, are summarized in Table 3.

**Table 3:** GO analysis of the corresponding differentially expressed proteins.

Protein function categories	Up-regulated in fibrosis model group	Up-regulated in rhCygb treatment group
Response to oxidative stress	Pink1; 60 kDa heat shock protein, mitochondrial	Apolipoprotein E; Peroxiredoxin-2; Hbb-b1 hemoglobin, beta adult major chain; Hemoglobin subunit alpha-1/2
Response to stimulus	Argininosuccinate lyase; FGFR1 oncogene partner 2 homolog	Peroxiredoxin-2; Beta-2-microglo-bulin; C4-2 complement component 4, gene 2
Regulation of immune system process		Complement C4; Beta-2-microglobulin; Ig lambda-2 chain c region; Ig kappa chain c region, B allele; Ifi441
Response to inflammtion	60 kDa heat shock protein, mitochondrial	Apolipoprotein E; C4-2 complement component 4, gene 2; Complement C4
Oxygen transpotation		Hemoglobin subunit alpha-1/2; Hbb-b1 hemoglobin, beta adult major chain; Galectin-5; Galectin-9
NF-kB signaling	Proteasome subunit beta type-6; Clusterin; Pink1	

## Discussion

In the present study, we used CCl4 to establish a liver fibrosis model[2, 21, 22], as confirmed by histological evaluation and the increased levels of the Serum parameters ALT, AST, HA, LN, Col III and Col IV in the model group, compared with the control group. After treatment with rhCygb, the levels of serum markers were decreased, compared with the fibrosis model group, and the livers of rats treated with rhCygb showed a reduction in liver injury, exhibiting a clear improvement. This suggests that rhCygb may play a role in a reversion of the progression of liver fibrosis, but the anti-fibrotic molecular mechanism remains under investigation. Experimental evidence and gene annotation showed that Cygb has many functions, including O2 storage and diffusion, which reduce oxidative stress, and sensing for cellular respiration and metabolism, NO scavenging and peroxidase activity, which protect cells against oxidative damage by free radical species. High-quality sample preparation prior to 2-DE is critical for producing meaningful results. To enhance detection of low-abundance serum proteins in 2-DE gels, we experimented with and compared the ProteoMiner kit[20] and the Albumin/IgG removal kit for removing the high-abundance proteins. As shown in Fig 2 and evidenced by the superior separation of analytes, protein enrichment and detection of low abundance proteins were far greater using the ProteoMiner kit than using the Albumin/IgG removal kit. Finally, we utilized the optimized conditions of 2-DE coupled with MALDI-TOF-MS analysis to obtain 43 spots and identify 30 proteins of interest.

We analyzed the differentially expressed proteins and, taking into consideration their functional annotation, identified several proteins that may be related to the molecular mechanism of the anti-fibrotic effect of rhCygb treatment. Several proteins were down-regulated in the fibrosis model samples, yet were detected at high levels in the control and rhCygb treated groups. For example, hemoglobin subunit alpha -1/2 and hemoglobin subunit beta-1, which are directly involved in O2 transport [6], and galectin-5 and galactin-9, which enhance O2 transport by their participation in red blood cell differentiation, were under-expressed in the fibrosis model rats. Likewise, the anti-oxidant peroxiredoxin-2[23] was also found to be down-regulated in the fibrosis model samples. Proteins related to immune system processes, including Ig kappa chain c region, beta-2-microglobulin, Ifi441 and complement C4, were also found to be under-expressed in rats in the fibrosis model group. Previous reports have indicated that complement C4 could be a biomarker of liver fibrosis[24, 25]. Additionally, some proteins were found to be over-expressed in the fibrosis model group, including acute phase proteins such as 60 kDa heat shock protein (HSP60), FGFR1 oncogene partner 2 homolog and clusterin. Rats given chronic treatments of high concentrations of CCl4 suffered liver tissue injury and necrosis. As part of the inflammation response, HSP60 was over-expressed to resist the adverse external stimulus. FGFR1 oncogene partner 2 homolog and clusterin, which are involved in the wound response[26], were similarly up-regulated. However, apolipoprotein E, which is a negative feedback inhibitor of the inflammatory response, was under-expressed in fibrosis model group. Among the proteins we identified and analyzed, proteasome subunit beta type-6, clusterin and pink1 which were over-expressed in the fibrosis model group are involved in NF-κB signaling and are related to the initiation and regulation of immune and inflammatory responses, cell proliferation and tumorigenesis[27, 28]. The biological functions of the differentially expressed proteins relate mainly to oxidative stress[6, 29], immune and inflammatory responses, and NF-κB signaling and give an insight into the underlying molecular mechanisms of the anti-fibrotic effect of rhCygb in the treatment of rats liver fibrosis. Given that liver biopsy is an invasive and expensive method, often poorly tolerated by patients, the use of easily obtained serum or plasma is a desirable choice for biomarker studies to improve the diagnosis of liver fibrosis patients through serum level detection.

## Conclusion

In conclusion, the present study demonstrates that the effect of rhCygb on CCl4-Induced hepatic fibrogenesis in rat. The rhCygb next study may be an original candidate drug in the treatment and reversal of liver fibrosis. However, further elucidation of the molecular roles played by the differentially expressed proteins of interest is necessary. Additionally, larger scale studies using human specimens will be required to further clarify the specific role of Cygb in hepatic fibrosis.

## References

[1] Toosi AE. Liver fibrosis: Causes and methods of assessment, a review. Rom J Intern Med. 2015;53: 304–314. doi: 10.1515/rjim-2015-0039 26939206 10.1515/rjim-2015-0039

[2] Shi C, Li G, Tong Y, Deng Y, Fan J. Role of CTGF gene promoter methylation in the development of hepatic fibrosis. Am J Transl Res. 2016;8: 125–132. 27069546 PMC4759422

[3] Trebicka J, Hennenberg M, Odenthal M, Shir K, Klein S, Granzow M, et al Atorvastatin attenuates hepatic fibrosis in rats after bile duct ligation via decreased turnover of hepatic stellate cells. J Hepatol. 2010;53: 702–712. doi: 10.1016/j.jhep.2010.04.025 20633948 10.1016/j.jhep.2010.04.025

[4] Ratib S, Fleming KM, Crooks CJ, Aithal GP, West J. 1 and 5 year survival estimates for people with cirrhosis of the liver in England, 1998–2009: A large population study. J Hepatol. 2014;60: 282–9. doi: 10.1016/j.jhep.2013.09.027 24128415 10.1016/j.jhep.2013.09.027

[5] Kawada N, Kristensen DB, Asahina K, Nakatani K, Minamiyama Y, Seki S, et al Characterization of a stellate cell activation-associated protein (STAP) with peroxidase activity found in rat hepatic stellate cells. J Biol Chem. 2001;276: 25318–25323. doi: 10.1074/jbc.M102630200 11320098 10.1074/jbc.M102630200

[6] Oleksiewicz U, Liloglou T, Field JK, Xinarianos G. Cytoglobin: Biochemical, functional and clinical perspective of the newest member of the globin family. Cell Mol Life Sci. 2011;68: 3869–3883. doi: 10.1007/s00018-011-0764-9 21744065 10.1007/s00018-011-0764-9 PMC11115184

[7] Motoyama H, Komiya T, Thuy LT, Tamori A, Enomoto M, Morikawa H, et al Cytoglobin is expressed in hepatic stellate cells, but not in myofibroblasts, in normal and fibrotic human liver. Lab Invest. 2014;94: 192–207. doi: 10.1038/labinvest.2013.135 24296877 10.1038/labinvest.2013.135

[8] Thuy LTT, Matsumoto Y, Thuy TTV, Hai H, Suoh M, Urahara Y, et al Cytoglobin deficiency promotes liver cancer development from hepatosteatosis through activation of the oxidative stress pathway. The American Journal of Pathology. 2015;185: 1045–1060. doi: 10.1016/j.ajpath.2014.12.017 25665792 10.1016/j.ajpath.2014.12.017

[9] Nakatani K, Okuyama H, Shimahara Y, Saeki S, Kim DH, Nakajima Y, et al Cytoglobin/STAP, its unique localization in splanchnic fibroblast-like cells and function in organ fibrogenesis. Lab Invest. 2004;84: 91–101. doi: 10.1038/sj.labinvest.3700013 14647402 10.1038/labinvest.3700013

[10] Tejero J, Kapralov AA, Baumgartner MP, Sparacino-Watkins CE, Anthonymutu TS, Vlasova II, et al Peroxidase activation of cytoglobin by anionic phospholipids: Mechanisms and consequences. Biochim Biophys Acta. 2016;1861: 391–401. doi: 10.1016/j.bbalip.2016.02.022 26928591 10.1016/j.bbalip.2016.02.022 PMC4821708

[11] Li Z, Wei W, Chen B, Cai G, Li X, Wang P, et al The effect of rhCygb on CCl4-Induced hepatic fibrogenesis in rat. Sci Rep-UK. 2016;6: 23508 doi: 10.1038/srep23508 27006085 10.1038/srep23508 PMC4804332

[12] Raatschen N, Bandow JE. 2-D gel-based proteomic approaches to antibiotic drug discovery. Curr Protoc Microbiol; 2012 pp. 1t1F–t2F. doi: 10.1002/9780471729259.mc01f02s26 22875564 10.1002/9780471729259.mc01f02s26

[13] Cowan ML, Rahman TM, Krishna S. Proteomic approaches in the search for biomarkers of liver fibrosis. Trends Mol Med. 2010;16: 171–183. doi: 10.1016/j.molmed.2010.01.006 20304704 10.1016/j.molmed.2010.01.006

[14] Yang L, Rudser KD, Higgins L, Rosen HR, Zaman A, Corless CL, et al Novel biomarker candidates to predict hepatic fibrosis in hepatitis c identified by serum proteomics. Digest Dis Sci. 2011;56: 3305–3315. doi: 10.1007/s10620-011-1745-4 21590334 10.1007/s10620-011-1745-4 PMC3181275

[15] Lee JE, Lee JY, Kim HR, Shin HY, Lin T, Jin DI. Proteomic analysis of bovine pregnancy-specific serum proteins by 2D fluorescence difference gel electrophoresis. Asian Austral J Anim. 2015;28: 788–795. 10.5713/ajas.14.0790 PMC4412975 25925056

[16] Westermeier R, Gorg A. Two-dimensional electrophoresis in proteomics. Methods Biochem Anal. 2011;54: 411–439. 21954788 10.1002/9780470939932.ch17

[17] Cugno G, Parreira JR, Ferlizza E, Hernández-Castellano LE, Carneiro M, Renaut J, et al The goat (Capra hircus) mammary gland mitochondrial proteome: A study on the effect of weight loss using Blue-Native PAGE and Two-Dimensional gel electrophoresis. Plos One. 2016;11: e151599. 10.1371/journal.pone.0151599 PMC4816393 27031334

[18] Roche S, Tiers L, Provansal M, Seveno M, Piva M, Jouin P, et al Depletion of one, six, twelve or twenty major blood proteins before proteomic analysis: The more the better? J Proteomics. 2009;72: 945–951. doi: 10.1016/j.jprot.2009.03.008 19341827 10.1016/j.jprot.2009.03.008

[19] Cheng H, Hsieh S, Sung C, Pai BC, Liu N, Chen CP. Optimizing human bile preparation for Two-Dimensional gel electrophoresis. Biomed Res Int. 2016 doi: 10.1155/2016/5185317 26966686 10.1155/2016/5185317 PMC4757711

[20] Li L, Sun C, Freeby S, Yee D, Kieffer-Jaquinod S, Guerrier L, et al Protein sample treatment with peptide ligand library: Coverage and consistency. Journal of Proteomics & Bioinformatics. 2009;02: 485–494. doi: 10.4172/jpb.1000110

[21] Kataoka T, Yamato K, Nishiyama Y, Morii Y, Etani R, Takata Y, et al Comparative study on the inhibitory effects of alpha-tocopherol and radon on carbon tetrachloride-induced renal damage. Ren Fail. 2012;34: 1181–1187. doi: 10.3109/0886022X.2012.717496 22978362 10.3109/0886022X.2012.717496

[22] Chen S, Chen Y, Chen B, Cai Y, Zou Z, Wang J, et al Plumbagin ameliorates CCl4 -Induced hepatic fibrosis in rats via the epidermal growth factor receptor signaling pathway. Evid-Based Compl Alt. 2015 doi: 10.1155/2015/645727 26550019 10.1155/2015/645727 PMC4624924

[23] Han YH, Kim SU, Kwon TH, Lee DS, Ha HL, Park DS, et al Peroxiredoxin II is essential for preventing hemolytic anemia from oxidative stress through maintaining hemoglobin stability. Biochem Biophys Res Commun. 2012;426: 427–432. doi: 10.1016/j.bbrc.2012.08.113 22960070 10.1016/j.bbrc.2012.08.113

[24] Gangadharan B, Antrobus R, Dwek RA, Zitzmann N. Novel serum biomarker candidates for liver fibrosis in hepatitis C patients. Clin Chem. 2007;53: 1792–1799. doi: 10.1373/clinchem.2007.089144 17702858 10.1373/clinchem.2007.089144

[25] Sopena B, Martinez-Vazquez C, Fernandez-Rodriguez CM, de la Fuente J, Rivera A, Rodriguez M, et al Serum angiotensin converting enzyme and C4 protein of complement as a combined diagnostic index in alcoholic liver disease. Liver. 1996;16: 303–308. 8938630 10.1111/j.1600-0676.1996.tb00750.x

[26] Gafencu AV, Robciuc MR, Fuior E, Zannis VI, Kardassis D, Simionescu M. Inflammatory signaling pathways regulating ApoE gene expression in macrophages. J Biol Chem. 2007;282: 21776–21785. doi: 10.1074/jbc.M611422200 17553793 10.1074/jbc.M611422200

[27] Jimi E, Fukushima H. NF-kappaB signaling pathways and the future perspectives of bone disease therapy using selective inhibitors of NF-kappaB. Clin Calcium. 2016;26: 298–304. 26813510

[28] Wang JY, Guo JS, Li H, Liu SL, Zern MA. Inhibitory effect of glycyrrhizin on NF-kappaB binding activity in CCl4- plus ethanol-induced liver cirrhosis in rats. Liver. 1998;18: 180–185. 9716228 10.1111/j.1600-0676.1998.tb00147.x

[29] Latina A, Viticchiè G, Lena AM, Piro MC, Annicchiaricopetruzzelli M. ΔNp63 targets cytoglobin to inhibit oxidative stress-induced apoptosis in keratinocytes and lung cancer. Oncogene. 2015;35: 1493–1503. doi: 10.1038/onc.2015.222 26096935 10.1038/onc.2015.222

